# Urban green space cooling effect in cities

**DOI:** 10.1016/j.heliyon.2019.e01339

**Published:** 2019-04-08

**Authors:** Farshid Aram, Ester Higueras García, Ebrahim Solgi, Soran Mansournia

**Affiliations:** a Escuela Técnica Superior de Arquitectura, Universidad Politécnica de Madrid-UPM, Madrid, 28040, Spain; b School of Engineering and Built Environment, Griffith University, Australia; c Faculty of Architecture and Urbanism, University of Art, Tehran, Iran

**Keywords:** Energy, Environmental science

## Abstract

Urban green spaces are considered an appropriate way to reduce urban heat island effects and provide comfort to the nearby occupants. In addition to cooling the actual space, urban green spaces are also able to influence the surrounding area, and this phenomenon is called the urban green space cooling effect. The most important issues with regard to the cooling effects of urban green spaces are the intensity and density of the cooling, which can play a major role for urban designers and planners in dealing with urban heat island. This article reviews the latest studies that have examined the cooling effects of urban green spaces in recent years. Based on the method of evaluation of their samples, the studies are divided into three groups. The first category consists of research into a set of urban green spaces in one part of or in an entire city, mainly conducted through remote sensing and satellite maps. The second category investigates city parks or several urban parks with recognizable shapes and locations. In this section, information was mainly gathered by virtue of field observations. The third category relates to studies in which a part of urban space according to different scenarios of green space placement was modeled by simulation. The results of the present study illustrate that the highest cooling effect distance and cooling effect intensity are for large urban parks with an area of more than 10 ha; however, in addition to the area, the natural elements and qualities of the urban green spaces, as well as climate characteristics, highly inform the urban green space cooling effect.

## 1 Introduction

The intensified accumulation of greenhouse gases in the Earth's atmosphere has led to rapid changes in global temperature trends and climate (IPCC, 2017; Ng and Ren, 2017). While disrupting our daily lives and causing financial losses, these changes are further expected to have serious safety, security, and health implications (Demuzere et al., 2014; Heltberg et al., 2008; Ebi and Paulson, 2007; Khanian et al., 2018). In urban areas, this issue has been exacerbated by another modern phenomenon called the Urban Heat Island (UHI) effect (Taha et al., 1988; Oke, 1982). Owing to UHI effect, an urban area can be on average 1.0–6.0 °C warmer than the nearby non-urban regions (Dimoudi et al., 2013). For example, USGCRP (2017) reported that because of UHI effect, American cities experience 0.5–4.0 °C higher daytime air temperatures and 1.0–2.5 °C higher night-time air temperatures than the nearby rural areas.

The UHI effect has several causes, including the increased absorption of sunlight by dark-colored surfaces of buildings, the physical properties of the materials commonly used in urban areas, the imposition of heat in the urban space due to the urban morphology which affects shading and air movement, the urban compactness which stems from density, plot ratio, land-use and travel proximity, and the deficiency in urban green spaces (Giridharan and Emmanuel, 2018; O'Malley et al., 2015; Kaloustian and Diab, 2015; Xi et al., 2012; Priyadarsini et al., 2008). This phenomenon is also exacerbated by the growing size of the city dwelling human population and the increasing rate of energy consumption (Mirzaei, 2015; Battista et al., 2016). Research has shown that cities currently account for 60–80% of the world's total energy consumption (Kamal-Chaoui and Roberts, 2009). It was also indicated that distance from UHI is a key factor affecting heating and cooling loads and thus the effect of urbanization on energy demand (Kolokotroni et al., 2010). Since it has been estimated that the ratio of world urban population to total population is set to increase from 54% in 2016 to 60% in 2030 (UN, 2016), UHI can be expected to become a major challenge in the future urban life.

There is an extensive body of literature on the available strategies to reduce the UHI effect (Aflaki et al., 2017; Rosenfeld et al., 1998; Azevedo and Leal, 2017; EPA, 2008; Wang et al., 2016; Huang et al., 1990). In general, the methods currently available for this purpose can be categorized into four groups: the use of vegetation cover like trees, shrubs and lawns at different scales (Gago et al., 2013; Mackey et al., 2012), the stack night ventilation (Kolokotroni et al., 2006), the use of waterbodies (Gunawardena et al., 2017; Moyer and Hawkins, 2017; Daniel et al., 2018), and the use of materials with high albedo rating for pavement and other ground surfaces (Pacheco-Torgal, 2015; Santamouris, 2013; Li et al., 2013a,b; Taha et al., 1988).

The effectiveness of green infrastructure in reducing urban thermal islands is already proven through measurements (filed measurements, scale models, and thermal remote sensing), and computer simulation (Farhadi et al., 2019; Lai et al., 2019; Zölch et al., 2016; Norton et al., 2015; Chow and Brazel, 2012; Wong and Yu, 2005). The literature related to this subject consists of broad investigations into green infrastructure of different shapes and scales (Wang and Banzhaf, 2018), including small local parks (Ca et al., 1998; Aram et al., 2019), large urban parks (Petralli et al., 2009; Buyadi et al., 2015), urban forests (Oke et al., 1989; Brandt et al., 2016), urban gardens (Mazhar et al., 2015), green roofs (Santamouris, 2014; Alcazar et al., 2016; Besir and Cuce, 2018), green facades (Demuzere et al., 2014; Manso and Castro-Gomes, 2015), and street trees (Lobaccaro and Acero, 2015; Shahidan et al., 2010; Klemm et al., 2015).

The majority of investigations into the effect of features and dimensions of urban green spaces (UGSs) on UHI have been conducted over the past ten years (Akbari and Dionysia, 2016). According to a review study published in 2010 (Bowler et al.), green infrastructure (trees, parks, forests, and green roofs) have a higher level of thermal comfort than other urban spaces. This is especially true for larger parks and urban forests (UGS), which can have up to 0.94 °C lower daytime temperatures. Another recent review study has shown that thermal comfort and the UHI reduction effect of a UGS depends on its size and shape. According to this study, the cooling effect of an UGS is directly correlated with its vegetation cover and tree shade area (Jamei et al., 2016). In a recent review paper by Taleghani (2018), among the strategies for reducing the effect of UHI, the role of effective UGSs has been emphasized. By taking six Urban Parks Studies (UGS) into account, it has also been demonstrated that these spaces play a major role in UHI reductions.

Hence, thanks to their vast area and diverse vegetation cover, urban parks have a much more significant cooling and thermal comfort impact than small green spaces (Givoni, 1991). As a result, these parks have become known as effective countermeasures against the UHI effect. In the urban studies and sustainability literature, the cold aura around urban parks has become known as “Park Cool Island” (PCI) (Cao et al., 2010; Spronken Smith and Oke, 1998) and lately, as “Green Space Cool Island” (GCI) (Martins et al., 2016; Du et al., 2017). A recent study by Bartesaghi Koc et al. (2018) showed that from all the studies conducted on the cooling effect of green infrastructure, the contribution of the cooling effect of parks and UGSs was 10.9% (PCIs: 6.7% and GCIs 4.2%). PCI or GCI generally refers to the cooling impact of an UGS not only on the area within the park but on the surrounding area as well. Modern development planning science considers the UGS cooling effect to be a highly effective solution for dealing with thermal islands.

Although the growing public and academic attention to UHI has encouraged research into this subject, the studies and findings in relation to the cooling effect of UGSs are yet to be summarized in a review. While there are some review studies on the cooling effect of urban parks, they are not specific and also cover other green infrastructure such as street trees, green roofs and green facade (Bowler et al., 2010; Jamei et al., 2016; Taleghani, 2018). The present study aims to review and categorize the recent studies carried out regarding the relation of characteristics of UGSs to their cooling effect, in order to facilitate the study of data collection and analysis methods commonly used in this area, and thus assist future planning and development attempts to use urban parks and urban gardens for creating the cooling effect and countering the UHI effect.

## 2 Main text

### 2.1 Research methodology

This paper is a systematic review of recent research on the utilization of UGSs for creating a cooling effect. Initially, a search was conducted for all articles that discuss urban greening at any scale with the purpose of creating thermal comfort and a cooling effect. Among these articles, those that included case studies on UGSs, in turn including parks, gardens and local green spaces were shortlisted. Since one section of the latest review study on the impact of green spaces on UHI (2010) (Bowler et al.) was dedicated to urban parks and green spaces, the articles published before 2010 were excluded. The remaining recent articles were first studied in greater detail.

Studies on the relation of UGSs to their cooling effect can be classified into three categories: (i) studies that investigate the combined impact of a group of UGSs and provide no specific information about the characteristics of individual green spaces; (ii) studies that contain specific information about the region and location of the cases studied; (iii) studies in which the cooling effect of UGSs has been examined using computer simulation based on several scenarios regarding the specifications of the green space. After reorganizing the data into the aforementioned categories, articles in the same category were compared in terms of quantitative and qualitative data and findings, including the number, shape, size, and dimensions of green spaces, the type of vegetation cover, and the resulting cooling effect. Consequently, the results of this review will be presented in three sections.

### 2.2 Cooling effect of a group of urban green spaces

In this section, the studies that have investigated the combined impact of a group of UGSs on a certain part or the entire domain of a city (Table 1) are examined. In general, the objective of these studies is to determine how effective is a group of urban green spaces in reducing UHI and cooling the environment. Given the well-known utility of remote sensing methods in urban ecological studies (Wilson et al., 2003), most of the research in this category has employed satellite imagery data from sources such as LST map, QuickBird, IKONOS, and ASTER for convenient analysis of the effect of green spaces over large expanses. Some of the studies to be discussed in this section have also utilized field observations, temperature measurements, or temperature sensors for better examination of cooling effect intensity (CEI) and cooling effect distance (CED).

**Table 1:** Summary of studies investigating the cooling effect of a group of UGSs.

Ref		Location (Köppen and Geiger Climatic classification, Kottek et al., 2006)	Month	Green site & comparator	Features of green site	Size	Purpose	Methods/Instruments	Conclusion
Cao et al. (2010)		Nagoya, Japan (Cfa)	May 25, July 10, October 30	92 parks compared with the surrounding area	Trees, grass, shrubs, soil, water, low albedo surfaces, high albedo surfaces	0.1ha to 41.9 ha	Identifying the role of park parameters (e.g., park size, land-use types, and shapes) in the PCI phenomenon	• Remotely sensed ASTER LST data and IKONOS image • Multivariate Regression	Cooling effects rely on the park characteristics and seasonal radiation conditions. Also, trees, shrubs and compactness of park benefit the PCI in spring and summer.
Du et al. (2017)		Shanghai, China (Cfa)	February	68 green spaces	Including trees and shrubs, lawn, different buildings and water body	1.12 ha to 205.32 ha	Indicating the role of UGS for implementing cooling effect and distinguishing efficient relevant elements in CEI and CED	• LST image • ArcGIS version 10.1 • MATLAB 2014 • Pearson correlation	GCI impacts are contingent upon green space itself and its surrounding features. Furthermore, raising vegetation and water body fractions or reducing impervious surfaces helps to improve GCI impacts.
Lin et al. (2015)		Beijing, China (Dwa)	September 22	30 parks compared to city center	Trees and shrubs	18.42 km^2^ in total	Developing an alternative method for calculating the cooling extent of green parks by using remote sensing	• Remote sensing, LST map • Dry and wet bulb thermometers (24st)	The area around a park that benefits from the cooling effect increases with park size.
Yu et al. (2017)		Fuzhou, China (Cfa)	From January to July	435 green patches (connected, and disconnected with water bodies)	329 patches: tree-based (280) and grassland-based (49)	0.02 ha to 296.7 ha	Quantifying which form of greenspace has the greatest cooling effect: simple or complex shape, large or small areas	• LST map, ArcGIS • hierarchical cluster analysis (CLU)	Compact greenspaces in the shape of a circle or square provide significant cooling effects in terms of intensity and efficiency
Feyisa et al. (2014)		Addis Ababa, Ethiopia (Cwb)	October 4–18	21 green areas: public parks, green spaces around building and private parks.	Green areas with dense tree vegetation (canopy cover of at least 60%)	0.85–22.3 ha	Identifying the physical characteristics of USG which determine cooling efficiency and examining its extent of extension	• NDVI index • ArcGIS version 10.0 Regression model	Appropriate choice of species, geometry and size of parks may improve efficiency of urban cooling
Anjos and Lopes (2017)		Aracaju, Brazil (As)	July 19 to October 10	UGS around 7 urban climate stations in different parts of the city	Vegetated area: from 2.2% to 53% Water bodies: From 0 to 50%	Not mentioned	Assessing the UHI and PCI effects based on an urban climatological network	• Climatic sensor • Local Climate Zone map	Most UHI and PCI intensities do not develop only in the light winds and clear sky But these factor have remarkable impact
Brown et al. (2015)		Kuala Lumpur, Malaysia (Af); Lahore, Pakistan (Bsh); Alice Springs, Australia (Bwh); Kyoto, Japan (Cfa); Toronto, Canada (Dfb)	10 years data	Five different zones were compared	Five sites in five different climate zones	Various sizes	Recognizing the effect of microclimate modifications on thermal comfort caused by elements in the landscape	• Simulated by human thermal comfort model COMFA	Decreasing air temperatures through a ‘cool island park’ is a moderately effective strategy
Chang and Li (2014)		Taipei, Taiwan (Cfa)	August to September and December to February	60 urban parks were surveyed and compared with the surrounding area	Trees, shrubs and pavement	Various sizes	Exploring details related to the planning and design of city parks such that they may effectively cool surrounding urban areas	• Thermal sensor • Stationary regression method	Parks and other open spaces should be designed with less than 50% paved area and at least 30% trees, shrubs, and other shadings.
Chen et al. (2014)		Beijing, China (Dwa)	May 22, July 9, October 13 and November 14	Measured 6 types of UGS: wood-land, shrub land, grassland, cropland, rivers, lakes	UGS covering 35% of the overall study area	Total size: 6450 ha	Focusing on the effects of spatial patterns of urban green patches on their own surface cooling effect	• LST map & QuickBird (QB) image • Regression analyses	In addition to patch size, the other elements such as shape, edge or connectivity have cooling effects
Sun and Chen (2017)		Beijing, China (Dwa)	July 5 and July 29	Five types of UGS: Impervious land (IL), forest land (FL), grass land (GL), water body (WB), and bare land (BL).	Ringroad 5 of the city	Total size: 108.86 km2	Investigating the dominant combinations of landscape conversions (2012), and quantifying the change of mean LST	• ENVI software QuickBird (2002) and IKONOS (2012) • TM images	Greater focus on protecting natural forests in cities might provide greater benefits for climate mitigation.
Buyadi et al. (Dec 2013)		Shah-Alam, Malaysia (Af)	February 21 and January 21	Study site situated in center of the city with various kind of land use and green spaces	Water bodies, high dense trees, mixed vegetation	Total size: 8530 ha	Surveying the influence of development on UGS and UHI	• LST map • NDVI assessment	Decreasing the vegetation land cover in open spaces has a direct correlation to increasing UHI
Li et al. (2013a,b)		Beijing, China (Dwa)	September 8 and October 4	Seven landscape Metrics, based on easily calculated, interpretable, and little redundancy	Not mentioned	0.52 ha to 0.89 ha	Examining the effects of spatial resolution on the relationship between LST and the spatial pattern of greenspace	• ENVI 4.6 QuickBird, SPOT, and TM imagery • Pearson and partial Pearson correlation	The relationship between LST and the abundance of greenspace was negative, but with the spatial configuration of UGS varied by spatial resolution
Kong et al. (2014)		Nanjing, China (Cfa)	June 13	Part of the city includes the urbanized area of Nanjing and part of its suburbs	Impervious surface, water body, agricultural land, forest vegetation, and barren land	Total size: 9200 ha	Investigating the sensitivity of the cooling effect associated with greenspace to changes in scale;	• Correlation analyses • IKONOS image	CEI and characteristics formed by greenspace patterns, and increasing vegetation provide cooling effect
Zhang et al. (2014)		Beijing, China (Dwa)	June to August	6387 green space	Trees, shrubs, grass, tree-shrubs, shrub-grass	Total size: 22,556 ha	Measuring the ecological benefits of the cooling effect associated with the use of green spaces	• Empirical model (11 weather stations)	The cooling effect and the environmental benefits of UGS largely depend on the green space's structure and size
Mariani et al. (2016)		Milan, Italy (Cfb)	33 years data (1981–2014)	Five metropolitan sites	Different sites in various zones (rural, urban parks, sites located in canyons of the urban plateau, and urban peaks)	Not mentioned	Describing the behavior of the surface energy balance (SEB) and establishing a frequency distribution climatology of the sensible fraction (SF) index	• Weather stations and remotely sensed data • SEB model	The cooling effect of urban parks can be improved through ameliorating and optimizing single park structure components

The first article to be discussed in this category is a study conducted on 30 parks in Beijing, where it was found that the size of the parks has an impact on cooling effect creation. The parks investigated in this study had an average CED of between 85m and 284m, and could reduce the average temperature by about 2.3–4.8 °C (Lin et al., 2015). A more recent study on 435 green spaces in another city in China (Fuzhou) showed that CEI of a park is a function of not only its size, but also its shape and quality. This study found that circular or rectangular compact green spaces play a more significant role in cooling effect, and that the area of a green space and the diversity of its vegetation also directly affect the level of cooling. For example, they reported that the CED of a space with an area of 2.3 ha and ΔLST of 0.93 °C was 59.62m, but the CED of a space with an area of 35.78 ha and ΔLST of 4.43 °C reached as high as 279.19 m. The green spaces examined in this study had an average CEI of 1.78 °C and average CED of 104m (Yu et al., 2017). In a similar study, the impact of the geometric shape of 21 UGSs in Addis Ababa on the resulting cooling effect was investigated. This study found a negative relationship between CEI and Shape Index (SI), and a positive relationship between CED and SI, and park size. Among the cases investigated in this study, the one with great cooling impact had a CEI of 6.72 °C and a CED of 240m (Feyisa et al., 2014).

After studying 92 parks in Nagoya, Cao et al. (2010) found that PCI varies not only with physical factors but also with the season. They reported that in spring, summer and autumn, the largest park studied (41.9 hectares) had a peak CEI of 6.50 K, 6.82 K and 2.46K respectively, and the studied parks as a whole had an average CEI of 1.30 K, 1.16 K and 0.43K respectively. Ultimately, this study concluded that the highest cooling effect occurs in summer and autumn. However, a recent study conducted in Aracaju reported that in both hot and cold seasons, CEI remains within the range of 1.5–2.0 °C, though the intensity of UHI varies with season (Anjos and Lopes, 2017).

A recent analysis in 2017 of 68 green spaces found that in addition to areas and complex shapes that play an important role in creating the cooling effect, another factor termed water body, was also effective in CEI and CED indices. In this study, it was also found that among the green space areas surveyed (1.12 ha–205.32 ha), the area of 1.12 ha had the lowest CED and CEI which were respectively 90m and 0.78 °C; however, the area of 129.46 ha had the highest CED and CEI which were 1610 m and 9.35 °C, respectively (Du et al., 2017).

### 2.3 Cooling effect of particular urban green spaces with known specifications

This section discusses the articles where the location and characteristics (size and shape) of the studied UGSs are specifically mentioned. To enable more accurate examination of the cooling effect of green spaces, the articles in this category are further divided into three subcategories based on the size and type of the case studied: (i) large-scale urban parks with areas of more than 20 ha, (ii) medium-sized urban parks with areas of between 0.1 and 12 hectares; and (iii) local and small parks with areas of less than 0.1 hectares.

#### 2.3.1 Large-sized urban parks

Research in this subcategory has studied large urban parks mostly located in city centers (Table 2). The cooling effect of large urban parks has long been of paramount interest to urban planners (Almendros Coca, 1992; Ca et al., 1998). Thanks to their vast area and location in the heart of the city, these parks often have a significant impact on the temperature of urban spaces (Jauregui, 1990). The cooling effect of these parks is closely associated with their CED and CEI, which depends on several factors, including park size and shape, type and amount of vegetation cover, and regional climate.

**Table 2:** Summary of studies investigating the cooling effect of large-sized urban parks.

Ref	Location (Köppen classification, Kottek et al., 2006)	Month	Green site & comparator	Features of green site	Size	Purpose	Methods/Instruments	Conclusion
Hamada and Ohta (2010)	Nagoya, Japan (Cfa)	March and August	One park compared with urban areas	forest, lawn, ponds, fields, spaces containing monuments and badlands	147 ha	Clarifying the range of the cool-island effect of a green area on an urban area, as well as the relationship between vegetation and air temperatures	• with temperature and humidity sensors 24 fixed measurement sites	The range of the cooling effect as well as the relationship between the vegetation cover and air temperature throughout the year
Doick et al. (2014)	London, UK (Cfb)	August to December (nights)	One large park	Water body, mixed grass land and treed landscapes, and formal avenues and gardens	111 ha	Providing empirical evidence for the extent of cooling of London's UHI with one large greenspace	• Mobile measurement • A developed correlation	Using meteorological stations close to urban greenspace can lead to underestimation of urban heat island intensity due to the cooling effect of the greenspace.
Sun et al. (2017)	Beijing, China (Dwa)	August 21	One park, Comparison of entire park with uncovered sites in the park	Grass, 10 & 20 m trees, hardened ground, water body and buildings	102 ha	Assessing the impacts of these parameters on thermal comfort improving effect of UGSs.	• PET Index • Simulation by ENVI-met and Rayman • Regression method Stationary	The most significant influencing factor on the moderation of thermal comfort is the higher trees, while hardened ground exhibits a negative impact
Chen et al. (2015)	Shanghai, China (Cfa)	November to January	One park, The squares in the park were compared	Surrounded by trees and benches	21.42 ha	Examining the relationship between outdoor micro-meteorological conditions and people's thermal comfort perception	• PET index • Mobile micro meteorological stations	Visitors' thermal sensations and space use were more significantly affected by the micro- meteorological factors in winter compared with autumn
Mahmoud (2011)	Cairo, Egypt (Bwh)	December and June	One big park near the city center (compact urban fabric of the old city core)	9 different zones: peak, spine, entrance, fountain, lake, canopy, pavement,	26.01 ha	Assessing microclimatic and human comfort conditions in various zones within an urban park.	• Field measurement campaign (thermal index PET and TSV) • RayMan	The results of this study contribute to the practice of providing appropriate thermal comfort in urban parks to attract visitors in summer and winter seasons.
Buyadi et al. (Nov 2013)	Shah-Alam, Malaysia (Af)	Not mentioned	One big national park	Forest reserved and protected	1,507 ha	Investigating the effects of land use changes on the surface temperature of a big national park	• GIS • Remote sensing images	The vegetation areas can provide positive impacts on regulating high temperatures in urban areas.
Yan et al. (2018)	Beijing, China (Dwa)	Summer month	One big Central park	Park has hills, forest, lakes, wetland and other natural landscapes	680 ha	Investigating the cooling effect of big park on thermal environment of surrounding urban area	• Field measurement • Mobile micro meteorological stations	The cooling effect of the big park influences not only within the park but extends beyond the park's border.

A study conducted by Hamada and Ohta (2010) in Nagoya found that during summers, areas adjacent to Heiwa Park (147 ha) had up to 1.9 °C lower temperature than other areas. They reported that in summers, this park had a CED of 200–300 meters during night hours and 300–500 meters during day hours. In another study (Doick et al., 2014), the average night-time CED of Kensington Gardens (111 ha) in London over the period between August and December (5 months) was found to vary between 20 and 440 meters. According to this study, this park reduces the summer nighttime temperatures by an average of 1.1 °C and a maximum of 4 °C.

Besides CED and CEI, some studies of large parks have investigated the Physiological Equivalent Temperature (PET), which is an indicator of human comfort under temperature variations (Matzarakis et al., 1999; Matzarakis and Amelung, 2008), to measure the cooling effect of green spaces. A study conducted in Shanghai, China, showed that on a hot sunny day (August 21st, 2:00 pm), the *Yuan Dynasty Relics Park* (102 ha) decreased the PET by an average of 2 °C and a maximum of 15.6 °C (Sun et al., 2017). Another study conducted in Shanghai (Chen et al., 2015) reported that the cooling effect created by *Zhongshan Park* (21.42 ha) located in the city center resulted in a PET of 15–29 °C during winter. In a similar study by Mahmoud (2011), it was shown that during the hot months of summer, the cooling effect of *Cairo's central Park* (26.01 ha) results in a daytime PET of 22–30 °C and a nighttime PET of 21–29 °C.

#### 2.3.2 Medium-sized urban parks

The articles to be reviewed in this section (Table 3) can be divided into two groups: works where only a single park has been studied, and works where several parks of different sizes have been compared with each other. The articles belonging to the second group have utilized different criteria for comparison, most notably the park size, shape, location, and the type and quality of its vegetation cover (Bacci et al., 2002; Spronken Smith and Oke, 1998).

**Table 3:** Summary of studies investigating the cooling effect of medium-sized and small urban parks.

Ref	Location (Köppen and Geiger Climatic classification, Kottek et al., 2006)	Month	Green site & comparator	Features of green site	Size	Purpose	Methods/Instruments	Conclusion
Vaz Monteiro et al. (2016)	London, UK (Cfb)	June 20 to October 2, (nights)	8 Parks situated in central of city	Tree canopy (47%_174%) and grass (68%_91%)	0.2 ha to 12.1 ha	Modeling the extent of the local air cooling service and finding related greenspace area	• Geographic Information System (ArcMap 10 – Esri)	For the ranges of areas studied, the distance over which cooling is experienced increases linearly by increasing green areas. The relationships between cooling amount and areas are non-linear.
Cohen et al. (2012)	Tel Aviv, Israel (Csa)	June 15 to July 15 and January	10 sites: three urban parks, three street canyons, two urban squares and a lawn in the tissue of the city	Varied vegetation coverages (a variety of tree types and sizes, shrubs and lawn, and paved areas	2000 m2 to 3600 m2	Examining the diurnal and seasonal climatic behavior of green and bare urban spaces; and studying their impact on human thermal comfort	• Meteorological stations and relative humidity sensors • RayMan and PET calculations	The cooling effect caused by urban vegetation is much higher in summer than in winter and at midday than at nighttime.
Oliveira et al. (2011)	Lisbon, Portugal (Csa)	6 days of 2006 and 2007 (August and September)	One garden in densely urbanized area	Deciduous trees, small lakes, large trunks and well developed crowns predominate (85%)	0.24 ha	Investigating the thermal performance of a small green space and its influence on the weather parameters of the surrounding atmosphere	• Mobile measurement • RayMan	The thermal performance of green areas is contingent upon some factors, like the climatic envelop and locations of study areas.
Skoulika et al. (2014)	Athens, Greece (Csa)	July 29 to September 2	One park surrounded by a very dense area with medium size residential and commercial buildings	Covered by grass, various types of bushes, low trees (olives, acacias, etc.), dense medium and high size trees	60,000 m^2^	Understanding and analyzing the relative climatic conditions in the park compared to the reference urban areas, and evaluate its climatic contribution	• Field measurement (The nine fixed temperature and humidity stations) • Mobile sensors	The park has an important mitigation impact on its surroundings (3.3 K). Increased wind speeds increase the mitigation potential beyond the park limit
Park et al. (2017)	Seoul, South Korea (Dwa)	Aug 9, 16, 27 and Sep 6, 7, 11	6 Small green space within urban blocks	Different shape types: polygonal, linear, single, and mixed.	300 m^2^ to 650 m^2^	Understanding the cooling effect of SGs on urban block units based on their types (four types) and structures	• Choose district by either local climate zone (LCZ) model • Air temperature logger (Testo 174H)	Small green areas can bring a positive benefit by increasing the cooling effects in urban blocks, and configuration of green space (polygonal and mixed types) plays a more important role.

In a study conducted in London (Vaz Monteiro et al., 2016), eight city center parks with areas ranging from 0.2 ha to 12.1 ha were studied to determine the impact of park size on CEI and CED. In short, this study showed that green spaces with areas of 0.5–2 ha can only cause up to 0.3 °C temperature reduction over 40m distance, but the temperature reduction caused by green spaces with areas of 3–5 ha can extend over a 70–120 m distance and reach as low as 0.7 °C. It was also reported that larger green spaces with areas of up to 12.1 ha can decrease temperature by 1 °C over 180–330 m distances. In a study conducted in Tel Aviv (Cohen et al., 2012), the cooling effects of 10 urban parks with different sizes (0.2–0.36 ha) and different vegetation quality and diversity were compared. This study found that parks with dense vegetation cover have the greatest effectiveness in terms of cooling and thermal comfort. The greatest cooling effect was observed in summer, when the parks managed to reduce the temperature by up to 3.8 °C, resulting in a PET of 18 °C. In comparison, a smaller effect was observed in winter, when temperature reduction was 2 °C and the resulting PET was 10 °C.

Other studies on green spaces of medium-sized are focused on the cooling effect of a single park. In these works, comparisons have been made either between differently vegetated parts of a single park, or between the park as a whole and the surrounding spaces. Research on a 0.24 ha urban park in Lisbon showed that during hot summer days, the air within this park is up to 6.9 °C cooler than the surrounding area. This research highlighted other factors such as sunlight exposure, geometric shape, and wind speed as determinants of cooling effect (Oliveira et al., 2011). A similar study by Skoulika et al. (2014) on a 6 ha park in Athens reported that wind can have a significant impact on the magnitude of cooling effect. The CEI of this park was found to be between -0.7K and -8.8K during night hours, and between -0.2K and -2.6K during day hours.

#### 2.3.3 Small parks

Besides large and medium-sized parks, small parks can also play a role in creating a cooling effect. Generally, studies on the cooling effect of UGSs are more focused on large and medium-sized green spaces; however, among the articles in this area, in a study, the role of small parks is also mentioned. According to this study conducted by Park et al. (2017) in Seoul, small green spaces with an area of 300 m^2^ can result in 1 °C temperature reduction and slightly larger parks with an area of 650 m^2^ can reduce the temperature by up to 2 °C. This study found that the CEI of a park correlates with its size, and accurately predicted that a 1500 m^2^ green space would reduce the temperature by up to 3.6 °C. This study also showed that polygonal lands with combined vegetation cover can reduce the temperature by up to 4 °C (Table 3).

### 2.4 Cooling effect predicted in computer simulations

This section reviews the studies conducted more recently following the popularization of computer analysis, the use of ENVI-met and FLUENT software (CFD model), in urban biochemical studies (Table 4). The articles covered in this section have employed ENVI-met and CFD simulations to predict the cooling effect of green spaces with different shapes, dimensions, and placements in different scenarios.

**Table 4:** Summary of studies investigating the cooling effect predicted in computer simulations.

Ref	Location (Köppen and Geiger Climatic classification, Kottek et al., 2006)	Month	Simulated Factor	Simulated Item	Purpose	Methods/Instruments	Conclusion
Ng et al. (2012)	Hong Kong, China (Cfa)	May 9	different greenery percentages around high rise buildings	Green space within the city center with high building density	Establishing a compact site in the center consisting of mixed commercial and residential buildings	• Mobile meteorological station • ENVI-met • PET Index	Greening and more significantly tree planting must be positioned nearer to the area where human activities are concentrated
Skelhorn et al. (2014)	Manchester, UK (Cfb)	July 13	Five study areas with retail, office, and residential buildings surrounded by UGS	Vegetation, mature trees and new trees	Indicating the impact of greenspace types on temperature, and assessing the utility of ENVI-met in cooling effect	• ENVI-met • IButton temperature sensors • Radiation shields	UGS elements like mature trees have impact on the mitigation of high temperatures. On the other hand, asphalt has a significant negative effect
Lin and Lin (2016)	Taipei, Taiwan (Cfa)	July 2	Evaluation of 8 park spatial arrangement scenarios	simulating differing greenery percentages	Characterizing the influence of the spatial arrangement of urban parks on local temperature reduction.	• ENVI-met	A larger total park area, a greater number of parks, a greater area of the largest park, more evenly distributed park spaces, and more park diversity lead to more dramatic outdoor cooling effects
Middel et al. (2015)	Phoenix, USA (Bwh)	June 23	Eight simulation scenarios for the neighborhood	varying tree canopy cover, from 0% to 30% using a mix of native and non-native trees	Quantifying the thermal impact of two heat mitigation aspects: urban forestry, cool roof initiatives.	• ENVI-met	Increased tree coverage reduces air temperatures, but the magnitude of this impact, even with a 25% tree canopy cover, may not be sufficient to offset increased temperatures due to climate change.
Vidrih and Medved (2013)	Ljubljana, Slovenia (Cfb)	July	One urban park	Simulating different scenarios in 140m *140m area	Investigating the impact of the density and size (age) of trees, air temperatures and wind velocities on UGS	• Three-dimensional CFD modelling	The park cooling effect was determined according to local conditions on the surface of park elements on a selected extreme summer days.

One of the first works in which the cooling effect of green spaces was analyzed with ENVI-met was a study conducted in 2012, where the impact of replacing Hong Kong sidewalk pavements with green materials was simulated in various climatic scenarios. This study found that planting sidewalk trees in urban spaces result in a better cooling effect than building green surfaces such as green roofs (Ng et al., 2012). A similar study in Manchester also found that mature trees have a significant impact on the pavement surface temperature. The simulation results of this study showed that adding 5% mature tree density would reduce the surface temperature by 1.0 °C, and even adding 5% density saplings would result in 0.5 °C temperature reduction in urban areas (Skelhorn et al., 2014).

In another recent study (Lin and Lin, 2016), ENVI-met software was used to simulate eight scenarios regarding the placement of green spaces in Taipei City. Of these eight scenarios, three were dedicated to the placement of 6 hectares of green space, and the remaining five assessed the placement of 36 hectares of green space. In these simulations, the impact of park sizes and placement was studied. Ultimately, the results showed that the larger the size of the UGS, the greater will be the cooling effect. In this study, the best result in terms of cooling performance was achieved by using a combination of smaller parks placed alongside a larger green space.

Utilizing a three-dimensional CFD model in Ljubljana in Slovenia by Vidrih and Medved (2013), the study indicated that the summertime cooling effect of different parts of a 1.96 ha park is depended on its leaf area index (LAI). They also reported that in areas where LAI_sp_ (planting density of 45 trees with an age of 50 years, per hectare) is 3.16, CEI reaches -4.8 °C, but in the extremities of the park, where LAI_sp_ is 1.05, CEI reaches -1.2 °C.

### 2.5 Summary and discussion

The present paper reviewed the recent articles related to the impact of UGS on the creation of cooling effect, reduction of UHI, and provision of thermal comfort in urban environments. In the past ten years, the growing attention to the importance of green spaces, and especially parks, in the creation of cooling effect has led to the publication of many research works with different methodologies and at different scales in relation to this subject. Given the high number and variety of articles published on this subject, we categorized the works based on methodology and scale of research. Although the literature contains a number of review studies on the subject of urban parks and green spaces (Bowler et al., 2010; Jamei et al., 2016; Taleghani, 2018; Bartesaghi Koc et al., 2018), they have taken a comprehensive perspective and investigated the articles on the entirety of green infrastructure. Hence, in these studies, the research on urban parks and green spaces has been reviewed as a part of an extensively broader literature and there is no detailed categorization in regard to the methods and findings of the studies specifically focused on this subject. Considering this gap in the literature and the importance of UGS cooling effect for urban planning, we attempted to address the lack of a review study in literature in regard to UGSs and their impact on cooling effect.

In the first section of this paper, we reviewed the articles on the combined impact of a group of UGSs on a part or the entire area of a city. The studies covered in this section were mostly based on remote sensing methods, with the exception of a few works that utilized field observations as well.

**Fig. 1:**
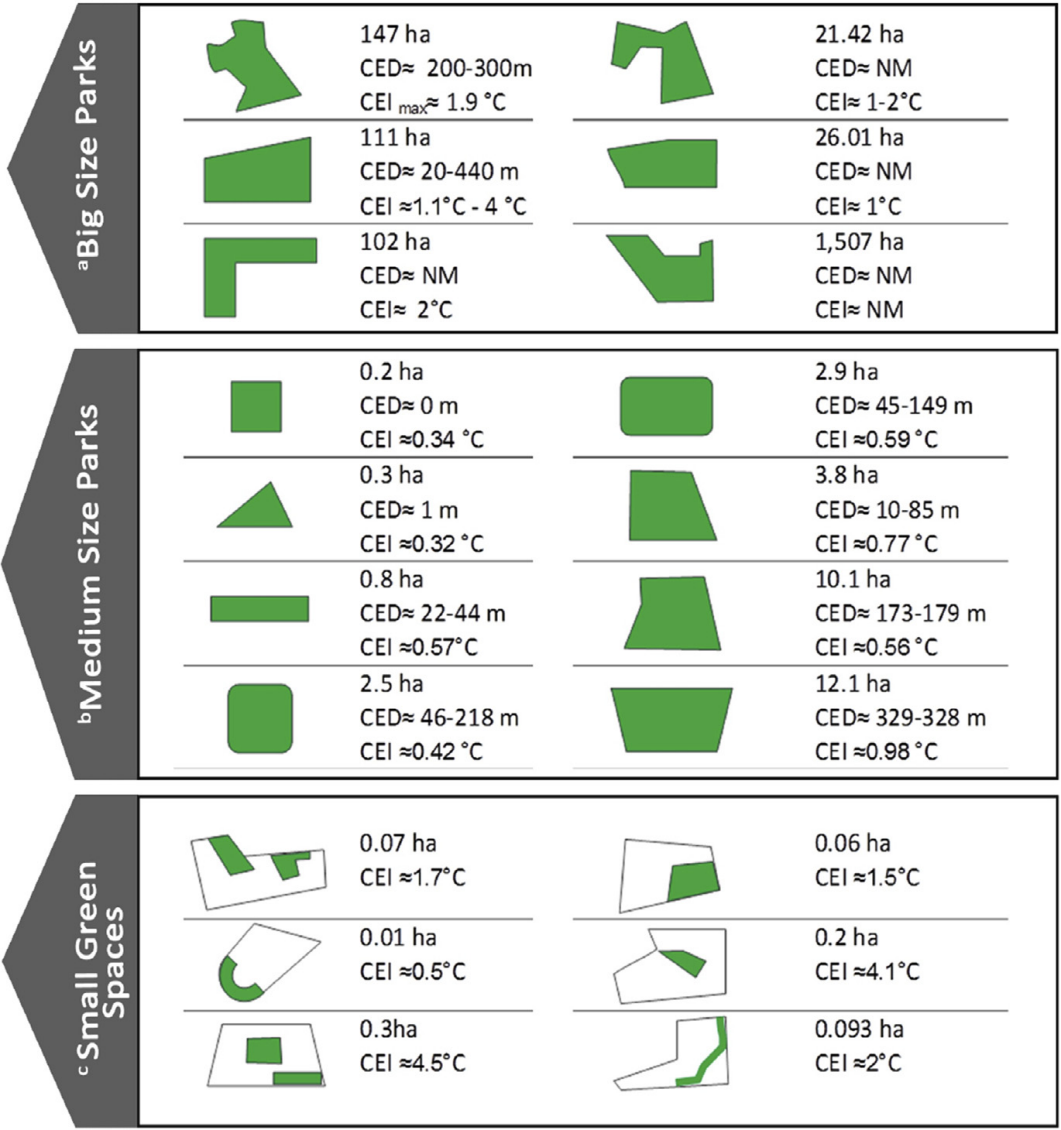
(a) 6 parks were investigated at this scale. (b) 21 parks were investigated at this scale. But, the information of Vaz Monteiro et al. (2016) study having both CEI and CED is illustrated. (C) 6 parks were investigated at this scale.

The next section of this study was dedicated to the articles where the location, size, and shape of the studied UGSs are specifically mentioned. These articles are mainly based on the studies of one or several specific parks, using field data collected from temperature sensors installed in and around the case, and/or by collecting PET data from residents to estimate the cooling effect. Some of these studies have also employed ENVI-met software, satellite imagery and remote sensing data for deeper analysis. For better categorization, these articles were divided into three subcategories based on the size of the studied case. A summary of results reported by these articles is presented in Table 5 and Fig. 1.

**Table 5:** Summary of studies investigating the cooling effect of particular UGSs with known specifications.

Size	General features
Big Size Parks	• Mature and tall trees with high percent of canopy • Water body • Different zones and landscapes with various vegetation types
Medium Size Parks	• Different size of trees (medium and high) • various vegetation types • small water body
Small Green Spaces	• Low tree diversity • Low vegetation diversity • Has an enclosure space

The last category of articles included the studies that have heavily utilized computer simulation. While being a relatively new method in this line of research, computer simulation has greatly facilitated the qualitative analyses that put more emphasis on the effect of vegetation type and quality and the placement of green spaces, rather than CED and CEI measurements. Although simulation is a convenient and quick method of analysis, it is not as accurate and reliable as the study of aerial maps and field observations, and is only suitable for developing initial hypotheses and assumptions and studying how the change of scenarios and variables affect the responses. As a result, the articles included in this section lack specific CED and CEI measurements. Overall, it can be concluded that each of the above methods has a set of unique features, which make it preferable for research at certain scales or with certain purposes.

Although the impact of UGS cooling effect has been researched at different scales and for different climates, the number and geographical distribution of studies in this field do not match the importance of the subject. The majority of past studies on this subject have been carried out in Eastern Asia, and their results cannot be generalized to other regions. Also, the majority of these studies are focused either on large and central parks or medium-sized parks, and have largely overlooked the smaller and local green spaces. Although it is known that larger parks have greater cooling effect, the only study that has investigated the effect of small parks (Park et al., 2017) has demonstrated the noticeable cooling effect of small parks on their surroundings, which indicates the necessity of further research into this matter.

It is also notable that the reviewed articles have overlooked some aspects of the subject in favor of a focus on the size of green spaces. While, some of these works have studied factors such as shape index (SI) (Feyisa et al., 2014), leaf area index (LAI) (Vidrih and Medved, 2013), wind speed (Skoulika et al., 2014), ΔLST of surfaces (Yu et al., 2017), seasonal changes (Anjos and Lopes, 2017), vegetation type (Cohen et al., 2012; Skelhorn et al., 2014; Middel et al., 2015), and sunlight exposure (Oliveira et al., 2011), none of them have examined the collective effect of that may influence the CED and CEI of a green space. In addition, most of these studies have ignored the role of natural and artificial objects and elements typically found within parks, such as waterbodies and urban furniture. A study on urban design and planning solutions to reduce the effects of urban heating was carried out by Kleerekoper et al. (2012) in the Netherlands, emphasizing on the use of various solutions, such as green space and waterbodies alongside each other. Furthermore, the other recent article that has considered the effect of the different qualitative and quantitative green space elements is the study of Xu et al. (2017), and there is still a huge gap in the research literature in regard to this issue.

Furthermore, only a few studies have considered the actual size of the vegetated area within green spaces. In other words, most studies, and especially those that have employed remote sensing methods, have assumed the entire area of green space (including sidewalks and buildings) as vegetated surface; a simplifying assumption that can undermine the accuracy of research. Another problem observed in the reviewed articles is the size mismatch between the studied cases, which may undermine the quality of comparisons, and thus the research power to discover the factors influencing the temperature reduction. In view of the above issues, it is imperative for future research to pay further attention to the specifications of the cases to be studied and to the selection of proper methodology according to research objectives and scale.

## 3 Conclusion

With steadily growing impacts of global warming, cities are increasingly struggling with new problems such as intensified UHI effect. Further academic attention to the impact of UGS on UHIs can provide city planners with viable strategies to address this issue. In this paper, we reviewed and categorized recent articles in this field of study. In summary, all of the reviewed articles agreed on the fact that UGSs, including parks, play a key role in reducing UHI, creating cooling, and providing thermal comfort for citizens.

In the reviewed articles, the impact of green spaces and their specifications were investigated through different approaches including the use of field observation and temperature sensors for accurate CEI and CED measurement and the use of satellite maps and remote sensing methods to investigate the collective impact of a group of UGSs on large expanses of a city. A good agreement was observed between the results derived from satellite imagery data and those obtained from field studies. From the results reported by these groups of research, it can be concluded that large parks with areas of more than 10ha have the highest average CED and CEI; that is, a 1–2 °C temperature reduction that extends over a 350m distance from the park boundary.

A relatively new method in this field of research is computer simulation, which allows research and analysis variables to be adjusted as desired. Given the unique merits and characteristics of the above methods and the success of previous attempts to combine field examinations with simulation or with remote sensing data, consideration of all these methods in line with research objectives and specifications can facilitate future research into cooling effect.

Since the majority of studies on cooling effect of UGS have been published in the last ten years, and this subject can be considered a relatively new branch of urban sustainability research, future studies are expected to cover broader geographic and climatic spectra and to focus their comparisons on carefully selected cases with similar characteristics such as size and shape. Future studies are also recommended to develop and test new advanced methods for this particular line of research and also incorporate the natural and artificial features commonly found in urban spaces into their analyses, in order to identify and evaluate the impact of all variables of UGS that play a role in temperature reduction through cooling effect.

## Declarations

### Author contribution statement

All authors listed have significantly contributed to the development and the writing of this article.

### Funding statement

This research did not receive any specific grant from funding agencies in the public, commercial, or not-for-profit sectors.

### Competing interest statement

The authors declare no conflict of interest.

### Additional information

No additional information is available for this paper
